# Impact of workplace bullying on job burnout and turnover intention among nursing staff in Greece: Evidence after the COVID-19 pandemic

**DOI:** 10.3934/publichealth.2024031

**Published:** 2024-04-29

**Authors:** Petros Galanis, Ioannis Moisoglou, Aglaia Katsiroumpa, Panayota Sourtzi

**Affiliations:** 1 Laboratory of Clinical Epidemiology, Department of Nursing, National and Kapodistrian University of Athens, Greece; 2 Department of Nursing, University of Thessaly, Greece; 3 Laboratory of Prevention, Department of Nursing, National and Kapodistrian University of Athens, Greece

**Keywords:** workplace, bullying, job burnout, turnover intention, nurses

## Abstract

**Introduction:**

The prevalence of workplace bullying, job burnout, and turnover intention among nursing staff increased during the COVID-19 pandemic. However, to the best of our knowledge, there are no studies that have measured the relationships among variables of interest after the pandemic.

**Objective:**

Our intention is to investigate the effect of workplace bullying on job burnout and turnover intention in nursing staff.

**Methods:**

We conducted a cross-sectional study during January–February 2024 in Greece. We obtained a convenience sample of 450 nurses. We used the 22-item Negative Acts Questionnaire-Revised to assess workplace bullying. We measured job burnout with the single-item burnout measure. We measured nurses' turnover intention with a valid 6-point Likert scale.

**Results:**

The study sample included 450 nurses with the mean age of 39.1 years (standard deviation [*SD*] = 10.2). The mean workplace bullying score was 7.7 (*SD* = 2.0), while the mean job burnout score was 7.7 (*SD* = 2.0). Among our nurses, 57.3% showed a high level of turnover intention. After eliminating confounders, we found that increased workplace bullying (adjusted beta = 0.031, 95% confidence interval [*CI*] = 0.023 to 0.039, *p* < 0.001) was associated with increased job burnout. Moreover, multivariable logistic regression analysis showed that increased turnover intention was more common among nurses who experienced higher levels of workplace bullying (adjusted odds ratio = 1.057, 95% *CI* = 1.043 to 1.071, *p* < 0.001).

**Conclusion:**

We found a positive relationship between workplace bullying, job burnout, and turnover intention. Nurse managers, organizations, and policy-makers ought to consider such findings to intervene and decrease workplace bullying by improving working conditions.

## 1. Introduction

Workplace bullying can be defined as a situation where a worker is, for a long time period, exposed to persistent negative mistreatment, consisting of frequent and constant criticism and person-related physical, verbal, or psychological violence [1],[2]. The three main characteristics of bullying, which differentiate it from some isolated episodes of conflict or workplace aggression, are that the employee becomes the target of repeated negative and undesirable social behaviors, the experience is for a lengthy period of time, and the victims perceive that they cannot easily escape from the negative situation or stop the undesirable behavior [3].

The healthcare sector is a workplace with a high prevalence of bullying, with up to 84% of health professionals reporting that they have been bullied at work [4],[5]. Moreover, the prevalence of bullying among nurses ranges from 2%–81% [6]. The lack of leadership skills and organizational support from supervisors, the highly stressful and demanding working environment of nurses, and the limited number of measures to prevent bullying may enhance the development of bullying behavior [7]–[9]. The pandemic created a particularly demanding working environment characterized by very high workloads, overtime, stress, burnout, and lack of material resources. Within these working conditions, incidents of bullying increased [10].

The phenomenon of burnout, which constitutes a response to prolonged exposure to occupational stressors, since the mid-1970s when it was first named in a study that examined the experience of people working in healthcare [11], continues to be the subject of many studies in the health sector even today. Among health professionals, nurses experience high rates of burnout. There are estimations that the prevalence of burnout worldwide is 11.23% [12], however studies in different settings and countries show that it exceeds 30% of nursing staff [13]–[15]. Maslach and colleagues have highlighted six organizational attributes that are related to the development of burnout in healthcare organizations: workload, control, reward, community, fairness, and values [11],[16]. Nurses working in understaffed departments, with high workloads and shortages of material resources, are more likely to develop burnout [17]–[19]. Studies in different settings reveal the high incidence of bullying, which is significantly associated with the development of burnout among nurses [20]–[22]. Also, when nurses' work organizations recognize their valuable contributions and show concern for their well-being, with nurses participating in hospital affairs and decision-making, as well as having good collegial nurse–physician relationships and organizational values for support, goals, innovation, and rules, then the likelihood of nurses experiencing burnout is reduced [23]–[26]. During the pandemic period, the same organizational factors were found to influence the occurrence of high rates of burnout in nursing staff [27]. However, even after the pandemic, burnout rates among nurses remain very high, showing that demanding working conditions and organizational weaknesses still exist [28].

A particularly frequent phenomenon among nursing staff, which affects the staffing and functioning of healthcare organizations, is their turnover intention. This may involve changing positions within the organization, moving to another, or even leaving permanently from the profession and following another career path [29]. Given that turnover intention is a strong predictor of the actual turnover behavior [30],[31], it is a constant threat than could affects the staffing and functioning of healthcare organizations. Both prior to and during the pandemic, the prevalence of turnover intention of nurses remained very high, exceeding 40% [32],[33], which is higher than for other health professionals [34]. The end of the pandemic also finds a large percentage of nurses (>40%) declaring their turnover intention [35]. Several organizational factors, such as reduced organizational support and understaffing, create the conditions for nurses' turnover intention [36]–[39]. When nursing staff experience increased burnout, dissatisfaction, stress, fatigue, and depression, the likelihood of choosing turnover increases [13],[40]–[44]. In addition to the above factors, which take a physical and mental toll on nurses, there is also the burden of workplace bullying, with nurses who experience it declaring their turnover intention [45]–[47]. Increased turnover rates of nursing staff have multifaceted effects on their health, nursing care quality, interpersonal relationships, workload and work environment, nurses' perceptions of responsibility, and the economic burden of recruiting new staff [48].

Until now, there are no studies in Greece measuring the relationship between workplace bullying, job burnout, and turnover intention in nursing staff. Moreover, we have not identified studies worldwide that measured the relationships between these variables post-pandemic. Thus, our aim was to investigate the influence of workplace bullying on job burnout and turnover intention in nursing staff following the pandemic. In our study, we focused only on workplace bullying that nurses experienced from superiors and colleagues, and not from other sources such as patients.

## 2. Materials and methods

### 2.1. Study design

We conducted a cross-sectional study during January–February 2024. We enrolled a convenience sample of nursing staff. Inclusion criteria were: (a) work in clinical settings, i.e., hospitals, (b) clinical experience at least for two years, and (c) good use of the Greek language. We developed an anonymous online version of our questionnaire with Google forms, which was posted in nursing groups through social media. Additionally, we performed face-to-face interviews with nurses who did not have social media accounts.

Assuming a low effect size (*f^2^* = 0.03) of workplace bullying on job burnout and turnover intention, the number of independent variables (one predictor [i.e., workplace bullying] and five confounders, a confidence level of 95%, and a margin of 5% error, the sample size was estimated at 436 participants.

### 2.2. Measurements

We used the 22-item Negative Acts Questionnaire-Revised (NAQ-R) to evaluate nurses' perceptions of their workplace bullying experience across the preceding six months [49]. The NAQ-R [49] determines the frequency that participants have faced bullying behaviors in the last six months. The NAQ-R measures perceived exposure to bullying from colleagues and superiors, and includes items such as “coworkers/superiors withholding information which affects your performance”, “being ignored or excluded from coworkers/superiors”, and “excessive monitoring of my work from coworkers/superiors”. Answers are on a 5-point Likert-type scale ranging from 1 (never) to 5 (daily). The total NAQ-R score is estimated by summing up the 22 items and ranges from 22 to 110 [49]. A higher score implies more frequent workplace bullying. The Greek version of the NAQ-R is a valid measure [50]. Cronbach's alpha was 0.963 in our study.

We used the single-item burnout measure in this study [51]. This instrument assesses burnout on a scale from 0 (not at all burnt-out) to 10 (extremely burnt-out). Higher values indicate increased job burnout. The validated Greek version was used [52].

We assessed nursing staff's turnover intention with the following question: “How often have you seriously considered leaving your current job?” [53]. Answers are on a 6-point Likert-type scale that ranges from 1 (never) to 6 (extremely often). Higher values indicate higher turnover intention. Values <3 indicate a low level of turnover intention; values ≥4 indicate increased turnover intention.

For demographic and job characteristics, we included: sex (female/male), age (in years), clinical experience (in years), shift work (no/yes), and understaffed department (no/yes). Shift work referred to nurses working in the night shift as well as in the morning shift.

### 2.3. Ethical considerations

The study protocol was submitted for approval from the Ethics Committee of the Department of Nursing of the National and Kapodistrian University of Athens (approval number 479, 10 January 2024). We conducted this study in accordance with the Declaration of Helsinki [54]. Individual data of nursing staff were not collected and informed consent was obtained.

### 2.4. Statistical analysis

We present categorical variables as numbers and percentages. Also, we present continuous variables as the mean, standard deviation (*SD*), median, and range. We used the Kolmogorov-Smirnov test to detect continuous variables' distribution. The NAQ-R score [49], job burnout score, and age followed normal distribution but not clinical experience. The NAQ-R score was the independent variable; job burnout and turnover intention were the dependent variables. We considered demographic and job characteristics as potential confounders. Since the job burnout score was a continuous variable, we performed univariate and multivariable linear regression analyses. In this case, we present unadjusted and adjusted beta coefficients, 95% confidence intervals (*CI*), and *p*-values. Given that turnover intention was a dichotomous variable, we performed univariate and multivariable logistic regression analyses, and we calculated unadjusted and adjusted odds ratios (*OR*), 95% *Ci*s, and *p*-values. By constructing multivariable regression models, we removed confounding by demographic and job variables. Statistical significance was considered at *p* < 0.05. We performed our analysis with the IBM SPSS 21.0 (IBM Corp. Released 2012. IBM SPSS Statistics for Windows, Version 21.0. IBM Corp., Armonk, NY, USA).

## 3. Results

### 3.1. Demographic and job characteristics

The study sample was comprised of 450 nurses. The mean age of nurses was 39.1 years (*SD* = 10.2), the median age was 39 years, and 86.7% were females. In our sample, 74.7% have been working in night shifts and 80.2% declared that they were working in understaffed departments. The mean years of clinical experience was 14.3 (*SD* = 10.3), while the median and range were 14 and 39 years, respectively. The demographic and job characteristics are shown in Table 1.

**Table 1.** Demographic and job characteristics of nurses (*N* = 450).

**Variables**	***N***	**%**
Sex		
Males	60	13.3
Females	390	86.7
Age (years)^a^	39.1	10.2
Clinical experience (years)^a^	14.3	10.3
Shift work		
No	114	25.3
Yes	336	74.7
Understaffed department		
No	89	19.8
Yes	361	80.2

Note: ^a^ *mean*, standard deviation.

### 3.2. Study measures

The mean score on the NAQ-R was 51.5 (*SD* = 20.5), ranging from 22 to 108 and a median value of 47. The mean job burnout score was 7.7 (*SD* = 2.0), ranging from 0 to 10 and a median value of 8, and 57.3% (*n* = 258) of nurses had increased turnover intention.

### 3.3. Regression analysis

After eliminating confounders, we found that increased workplace bullying (adjusted beta = 0.031, 95% *CI* = 0.023 to 0.039, *p* < 0.001) was associated with increased job burnout. Additionally, nurses who have been working in night shifts (adjusted beta = 0.694, 95% *CI* = 0.296 to 1.091, *p* = 0.001) and those who have been working in understaffed departments (adjusted beta = 0.748, 95% *CI* = 0.329 to 1.168, *p* = 0.001) experienced higher levels of job burnout. Linear regression analysis with job burnout as the dependent variable is shown in Table 2.

**Table 2.** Univariate and multivariable linear regression analysis with job burnout (continuous variable) as the dependent variable.

**Independent variables**	**Univariate model**		**Multivariable model**	
	**Unadjusted coefficient beta (95% *CI*)**	***p*-value**	**Adjusted coefficient beta (95% *CI*)^a^**	***p*-value**
Females *vs*. males	0.155 (-0.381 to 0.691)	0.570	0.035 (-0.450 to 0.519)	0.889
Age (years)	0.034 (0.016 to 0.052)	<0.001	0.039 (-0.002 to 0.079)	0.064
Clinical experience	0.032 (0.014 to 0.049)	<0.001	0.008 (-0.033 to 0.049)	0.715
Shift work (yes *vs*. no)	0.543 (0.127 to 0.959)	0.011	0.694 (0.296 to 1.091)	**0.001**
Understaffed department (yes *vs*. no)	1.120 (0.674 to 1.566)	<0.001	0.748 (0.329 to 1.168)	**0.001**
NAQ-R score	0.033 (0.025 to 0.042)	<0.001	0.031 (0.023 to 0.039)	**<0.001**

Note: Bold *p*-values indicate statistically significant associations in the multivariable model. *CI*: confidence interval. ^a^ *p*-value for ANOVA < 0.001; *R^2^* for the final multivariable model was 19.9%.

Multivariable logistic regression analysis identified that increased turnover intention was more frequent among nurses who experienced higher levels of workplace bullying (adjusted odds ratio = 1.057, 95% *CI* = 1.043 to 1.071, *p* < 0.001). Moreover, we found that increased turnover intention was 1.690 times more frequent among nurses who have been working in understaffed departments (adjusted odds ratio = 1.690, 95% *CI* = 1.005 to 2.845, *p* = 0.048). Logistic regression analysis with turnover intention as the dependent variable is shown in Table 3.

**Table 3.** Univariate and multivariable logistic regression analysis with turnover intention (dichotomous variable) as the dependent variable (reference category: low level of turnover intention).

**Independent variables**	**Univariate model**		**Multivariable model**	
	**Unadjusted odds ratio (95% *CI*)**	***p*-value**	**Adjusted odds ratio (95% *CI*)^a^**	***p*-value**
Females *vs*. males	1.303 (0.756 to 2.246)	0.341	1.081 (0.583 to 2.002)	0.805
Age (years)	1.002 (0.984 to 1.021)	0.823	1.026 (0.974 to 1.079)	0.335
Clinical experience	1.001 (0.983 to 1.019)	0.944	0.988 (0.938 to 1.040)	0.645
Shift work (yes *vs*. no)	1.637 (1.068 to 2.511)	0.055	1.647 (0.989 to 2.742)	0.055
Understaffed department (yes *vs*. no)	2.351 (1.465 to 3.775)	<0.001	1.690 (1.005 to 2.845)	**0.048**
NAQ-R score	1.057 (1.043 to 1.071)	<0.001	1.057 (1.043 to 1.071)	**<0.001**

Note: Bold *p*-values indicate statistically significant associations in the multivariable model. *CI*: confidence interval. ^a^ *R^2^* for the final multivariable model was 28.2%.

## 4. Discussion

This study showed the association of workplace bullying with burnout and turnover intention of nurses. As we mentioned above in detail, we measured workplace bullying that nurses experienced by colleagues and superiors. Two other factors, shift work and understaffing, were also found to influence the occurrence of burnout and turnover intention. Violence at the workplace of nurses is a common phenomenon, as nurses are often victims of violence from patients and their relatives. Young nurses who have just entered the profession are more often victims of bullying than older and more experienced nurses [7],[55]. Despite their limited work experience, young nurses who are victims of bullying may suffer burnout and other mental health disorders, and this will ultimately lead to turnover or leaving the nursing profession entirely [56].

Nursing staff's turnover intention is an ongoing risk to the sustainability of both the performance of health organizations and the healthcare system. Turnover intention places a financial burden on healthcare organizations. It is estimated that the cost of recruiting, training, and filling the vacancy caused by the turnover of a nurse can reach $88 thousand dollars [57],[58]. At the same time, staffing problems arise until the position is filled, with the remaining nurses having a higher workload and being burdened with more night and weekend shifts. Also, the turnover and shortage of experienced, university graduate nurses can affect the efficiency of healthcare organizations [59]. According to the findings of this study, understaffing and shift working are associated with the manifestation of burnout and turnover intention. Thus, we observe that a vicious cycle of nurses' burden is created, where they are trapped between the interaction of bullying, burnout, turnover intention, and understaffing, with victims being not only themselves but the patients (whose safety is compromised) and, consequently, the healthcare organizations. The impact of turnover intention also extends to health systems. Nursing staffing is the Achilles heel of health systems and will probably be their greatest challenge in the coming years. It was mentioned above that turnover intention is a robust predictor of the actual turnover behavior, with high percentages of nurses reporting their intention for turnover. A USA National Council of State Boards of Nursing study revealed a severe nursing personnel shortage and a crisis, where 100,000 nurses were recorded leaving the profession during the COVID-19 pandemic, with almost 900,000 nurses expressing their turnover intention until 2027 [60]. This situation is made even more ominous by estimates that by 2030 there will be significant shortages of health professionals, with nurses holding the highest proportion of shortages [61]. The incidents of bullying experienced by nurses contribute to increasing work stress and, at the same time, to the deterioration of the quality of their working life [62]. Nurses, in their attempt to protect themselves in difficult working conditions, although they remain in their position, opt for quiet quitting, which is a situation where they provide their minimum services, just enough to avoid being fired [63]. Bullying is a factor that triggers the occurrence of quiet quitting in nurses [64]. However, quiet quitting does not seem to be enough to stop turnover either, as those who choose it are more likely to experience turnover [35].

Bullying has a direct and indirect (through burnout) effect on the quality of care. Nurses who are bullied are more prone to errors during the provision of care [65] and patients may experience adverse events [66]. Exhausted nurses due to bullying express worse personal- and ward-level patient safety opinions [67]. Patients hospitalized in units with a high rate of nurse burnout have a greater risk of suffering from a harmful event, such as a pressure ulcer, fall, medication error, or surgical wound infection, as well as expressing dissatisfaction with the care provided [68],[69]. Patient safety is often at risk, and efforts to improve patient safety by health service organization managements have been ongoing for decades [70],[71]. Therefore, bullying and burnout are an alarming issue as they undermine efforts to improve patient safety, creating conditions for errors and negligence.

Efforts to prevent and reduce bullying should start with interventions that address attributes of the nurses' work environment that are linked with the incidence of bullying behaviors. Other interventions that can be implemented may include training activities, cognitive rehearsal, team-building, and sharing experiences of nurse managers. Training programs usually target detecting bullying behavior and perceiving the effects of this behavior, responding under stress, recognizing conflict resolution strategies, establishing a safe environment, and handling critical debates. Finally, the contribution of nurse supervisors in reducing bullying includes creating a healthy work environment with the main characteristics of collaboration, respect, effective interpersonal communication, collegiality, and mutual support between those entering the profession and senior nursing staff [72].

Our study had several limitations. We conducted an online survey through social media to collect our data. Also, we used a convenience sample. Despite achieving the minimum requirements for sample size, we cannot generalize our results since our sample was not representative of nurses in Greece. Thus, we cannot extend our findings to all nurses or all healthcare workers in Greece. Therefore, scholars should pay attention to the generalization of our results. Further research with random and stratified samples of nurses may reduce this bias. We used valid tools to measure workplace bullying, job burnout, and turnover intention. However, these tools are self-reported and, consequently, information bias is probable. Moreover, we measured job burnout and turnover intention with scales that were comprised of only one item. Also, our measurement methods were weak since they were based on uncontrolled reporting. Thus, further studies with more valid measurements should improve our knowledge. We eliminated several confounders on the correlation among bullying and burnout and turnover intention. However, further confounders can be eliminated in future studies. Because we conducted a cross-sectional study, it is not possible to conclude a causal relationship among workplace bullying, job burnout, and turnover intention. Longitudinal studies monitoring nurses' attitudes may reduce this bias. We examined, for the first time, the effect of workplace bullying on burnout and turnover intention in a sample of nurses after the pandemic; further studies may expand this research question by examining the mediating or/and moderating effect of job satisfaction, quiet quitting, and coping strategies.

## 5. Conclusions

Workplace bullying is an important form of violence with a high prevalence in healthcare settings, with nurses experiencing such behaviors very often. The impact of burnout is multifaceted and negatively affects staff and patients. The significant consequences of burnout and turnover intention on the functioning of healthcare organizations make it imperative that healthcare organization managements mobilize to ensure a healthy work environment that does not foster the development of bullying behaviors while ensuring their optimal diffusion by staff and supervisors.

## Use of AI tools declaration

The authors declare they have not used Artificial Intelligence (AI) tools in the creation of this article.

## References

[1] Purpora C, Cooper A, Sharifi C (2019). Workplace bullying and risk of burnout in nurses: A systematic review protocol. JBI Database System Rev Implement Rep.

[2] Einarsen S (2000). Harassment and bullying at work: A review of the scandinavian approach. Aggress Violent Behav.

[3] Nielsen MB, Einarsen SV (2018). What we know, what we do not know, and what we should and could have known about workplace bullying: An overview of the literature and agenda for future research. Aggress Violent Behav.

[4] Álvarez Villalobos NA, De León Gutiérrez H, Ruiz Hernandez FG (2023). Prevalence and associated factors of bullying in medical residents: A systematic review and meta-analysis. J Occup Health.

[5] La Torre G, Firenze A, Colaprico C (2022). Prevalence and risk factors of bullying and sexual and racial harassment in healthcare workers: A cross-sectional study in Italy. Int J Environ Res Public Health.

[6] Bambi S, Foà C, De Felippis C (2018). Workplace incivility, lateral violence and bullying among nurses. A review about their prevalence and related factors. Acta Bio Medica.

[7] Karatuna I, Jönsson S, Muhonen T (2020). Workplace bullying in the nursing profession: A cross-cultural scoping review. Int J Nurs Stud.

[8] Shorey S, Wong PZE (2021). A qualitative systematic review on nurses' experiences of workplace bullying and implications for nursing practice. J Adv Nurs.

[9] Wolf LA, Perhats C, Clark PR (2018). Workplace bullying in emergency nursing: Development of a grounded theory using situational analysis. Int Emerg Nurs.

[10] Serafin L, Kusiak A, Czarkowska-Pączek B (2022). The COVID-19 Pandemic increased burnout and bullying among newly graduated nurses but did not impact the relationship between burnout and bullying and self-labelled subjective feeling of being bullied: A cross-sectional, comparative study. Int J Environ Res Public Health.

[11] Maslach C, Schaufeli WB, Leiter MP (2003). Job burnout. Annu Rev Psychol.

[12] Woo T, Ho R, Tang A (2020). Global prevalence of burnout symptoms among nurses: A systematic review and meta-analysis. J Psychiatr Res.

[13] Shah MK, Gandrakota N, Cimiotti JP (2021). Prevalence of and factors associated with nurse burnout in the US. JAMA Netw Open.

[14] Gómez-Urquiza JL, De la Fuente-Solana EI, Albendín-García L (2017). Prevalence of burnout syndrome in emergency nurses: A meta-analysis. Crit Care Nurse.

[15] Pradas-Hernández L, Ariza T, Gómez-Urquiza JL (2018). Prevalence of burnout in paediatric nurses: A systematic review and meta-analysis. PLoS One.

[16] Montgomery A, Panagopoulou E, Esmail A (2019). Burnout in healthcare: The case for organisational change. BMJ.

[17] Macphee M, Dahinten VS, Havaei F (2017). The impact of heavy perceived nurse workloads on patient and nurse outcomes. Adm Sci.

[18] Ghavidel F, Fallahi-Khoshknab M, Molavynejad S (2019). The role of organizational factors in nurse burnout: Experiences from Iranian nurses working in psychiatric wards. J Family Med Prim Care.

[19] Bae SH (2021). Intensive care nurse staffing and nurse outcomes: A systematic review. Nurs Crit Care.

[20] Lang M, Jones L, Harvey C (2022). Workplace bullying, burnout and resilience amongst perioperative nurses in Australia: A descriptive correlational study. J Nurs Manag.

[21] Johnson J, Cameron L, Mitchinson L (2019). An investigation into the relationships between bullying, discrimination, burnout and patient safety in nurses and midwives: Is burnout a mediator?. Journal of Research in Nursing.

[22] Amini K, Miyanaji H, Din Mohamadi M (2023). Bullying and burnout in critical care nurses: A cross-sectional descriptive study. Nurs Crit Care.

[23] Matziari A, Montgomery AJ, Georganta K (2017). The relationship between organizational practices and values with burnout and engagement. Current Psychology.

[24] Moisoglou I, Yfantis A, Tsiouma E (2021). The work environment of haemodialysis nurses and its mediating role in burnout. J Ren Care.

[25] Mudallal RH, Othman WM, Al Hassan NF (2017). Nurses' burnout: The influence of leader empowering behaviors, work conditions, and demographic traits. Inquiry.

[26] Tang Y, Wang Y, Zhou H (2023). The relationship between psychiatric nurses' perceived organizational support and job burnout: Mediating role of psychological capital. Front Psychol.

[27] Galanis P, Vraka I, Fragkou D (2021). Nurses' burnout and associated risk factors during the COVID-19 pandemic: A systematic review and meta-analysis. J Adv Nurs.

[28] Galanis P, Moisoglou I, Katsiroumpa A (2023). Increased job burnout and reduced job satisfaction for nurses compared to other healthcare workers after the COVID-19 pandemic. Nurs Rep.

[29] Hayes LJ, O'Brien-Pallas L, Duffield C (2006). Nurse turnover: A literature review. Int J Nurs Stud.

[30] Ki J, Choi-Kwon S (2022). Health problems, turnover intention, and actual turnover among shift work female nurses: Analyzing data from a prospective longitudinal study. PLoS One.

[31] Griffeth RW, Hom PW, Gaertner S (2000). A meta-analysis of antecedents and correlates of employee turnover: Update, moderator tests, and research implications for the next millennium. J Manage.

[32] Labrague LJ, De los Santos JAA, Falguera CC (2020). Predictors of nurses' turnover intention at one and five years' time. Int Nurs Rev.

[33] Said RM, El-Shafei DA (2021). Occupational stress, job satisfaction, and intent to leave: nurses working on front lines during COVID-19 pandemic in Zagazig City, Egypt. Environmental Science and Pollution Research.

[34] Rotenstein LS, Brown R, Sinsky C (2023). The association of work overload with burnout and intent to leave the job across the healthcare workforce during COVID-19. J Gen Intern Med.

[35] Galanis P, Moisoglou I, Malliarou M (2024). Quiet quitting among nurses increases their turnover intention: Evidence from Greece in the post-COVID-19 era. Healthcare.

[36] Galanis P, Moisoglou I, Papathanasiou I V (2024). Association between organizational support and turnover intention in nurses: A systematic review and meta-analysis. Healthcare (Switzerland).

[37] Chen H, Li G, Li M (2018). A cross-sectional study on nurse turnover intention and influencing factors in Jiangsu Province, China. Int J Nurs Sci.

[38] Bruyneel A, Bouckaert N, Maertens de Noordhout C (2023). Association of burnout and intention-to-leave the profession with work environment: A nationwide cross-sectional study among Belgian intensive care nurses after two years of pandemic. Int J Nurs Stud.

[39] Arslan Yürümezoğlu H, Kocaman G, Mert Haydarİ S (2019). Predicting nurses' organizational and professional turnover intentions. Japan Journal of Nursing Science.

[40] Lee EK, Kim JS (2020). Nursing stress factors affecting turnover intention among hospital nurses. Int J Nurs Pract.

[41] Lee E, Jang I (2020). Nurses' fatigue, job stress, organizational culture, and turnover intention: A culture–work–health model. West J Nurs Res.

[42] Pang Y, Dan H, Jung H (2020). Depressive symptoms, professional quality of life and turnover intention in Korean nurses. Int Nurs Rev.

[43] Kim H, Kim EG (2021). A meta-analysis on predictors of turnover intention of hospital nurses in South Korea (2000–2020). Nurs Open.

[44] Labrague LJ, Gloe DS, McEnroe-Petitte DM (2018). Factors influencing turnover intention among registered nurses in Samar Philippines. Applied Nursing Research.

[45] Al Muharraq EH, Baker OG, Alallah SM (2022). The prevalence and the relationship of workplace bullying and nurses turnover intentions: A cross sectional study. SAGE Open Nurs.

[46] Xia G, Zhang Y, Dong L (2023). The mediating role of organizational commitment between workplace bullying and turnover intention among clinical nurses in China: A cross-sectional study. BMC Nurs.

[47] Wolf LA, Perhats C, Delao AM (2021). Validation of a grounded theory of nurse bullying in emergency department settings. Int Emerg Nurs.

[48] Sawada S, Takemura Y, Isobe T (2022). Perceived impact of nurse turnover on the organization: A Delphi study on managers of nursing. J Nurs Manag.

[49] Einarsen S, Hoel H, Notelaers G (2009). Measuring exposure to bullying and harassment at work: Validity, factor structure and psychometric properties of the Negative Acts Questionnaire-Revised. Work Stress.

[50] Kakoulakis C, Galanakis M, Bakoula-Tzoumaka C (2015). Validation of the Negative Acts Questionnaire (NAQ) in a sample of Greek teachers. Psychology.

[51] Hansen V, Pit S (2016). The single item burnout measure is a psychometrically sound screening tool for occupational burnout. Health Scope (Zahedan).

[52] Galanis P, Katsiroumpa A, Vraka I (2023). The single item burnout measure is a reliable and valid tool to measure occupational burnout. medRxiv.

[53] Spector PE, Dwyer DJ, Jex SM (1988). Relation of job stressors to affective, health, and performance outcomes: A comparison of multiple data sources. J Appl Psychol.

[54] Association WM (2013). World medical association declaration of Helsinki: Ethical principles for medical research involving human subjects. JAMA.

[55] Smith CR, Palazzo SJ, Grubb PL (2020). Standing up against workplace bullying behavior: Recommendations from newly licensed nurses. J Nurs Educ Pract.

[56] Anusiewicz C V., Shirey MR, Patrician PA (2019). Workplace bullying and newly licensed registered nurses: An evolutionary concept analysis. Workplace Health Saf.

[57] Bae SH (2022). Noneconomic and economic impacts of nurse turnover in hospitals: A systematic review. Int Nurs Rev.

[58] Roche MA, Duffield CM, Homer C (2015). The rate and cost of nurse turnover in Australia. Collegian.

[59] Moisoglou I, Galanis P, Meimeti E (2019). Nursing staff and patients' length of stay. Int J Health Care Qual Assur.

[60] NCSBN (2023). NCSBN Research projects significant nursing workforce shortages and crisis | NCSBN, 2023.

[61] Boniol M, Kunjumen T, Nair TS (2022). The global health workforce stock and distribution in 2020 and 2030: A threat to equity and ‘universal’ health coverage?. BMJ Glob Health.

[62] Galanis P, Moisoglou I, Katsiroumpa A (2024). Association between workplace bullying, job stress, and professional quality of life in nurses: A systematic review and meta-analysis. Healthcare.

[63] Galanis P, Katsiroumpa A, Vraka I (2024). Nurses quietly quit their job more often than other healthcare workers: An alarming issue for healthcare services. Int Nurs Rev.

[64] Galanis P, Moisoglou I, Katsiroumpa A (2024). Impact of workplace bullying on quiet quitting in nurses: The mediating effect of coping strategies. Healthcare (Basel).

[65] Johnson AH, Benham-Hutchins M (2020). The influence of bullying on nursing practice errors: A systematic review. AORN J.

[66] Arnetz JE, Neufcourt L, Sudan S (2020). Nurse-reported bullying and documented adverse patient events: An exploratory study in a US hospital. J Nurs Care Qual.

[67] Johnson J, Cameron L, Mitchinson L (2019). An investigation into the relationships between bullying, discrimination, burnout and patient safety in nurses and midwives: Is burnout a mediator?. Journal of Research in Nursing.

[68] Kakemam E, Chegini Z, Rouhi A (2021). Burnout and its relationship to self-reported quality of patient care and adverse events during COVID-19: A cross-sectional online survey among nurses. J Nurs Manag.

[69] Jun J, Ojemeni MM, Kalamani R (2021). Relationship between nurse burnout, patient and organizational outcomes: Systematic review. Int J Nurs Stud.

[70] Schwendimann R, Blatter C, Dhaini S (2018). The occurrence, types, consequences and preventability of in-hospital adverse events - A scoping review. BMC Health Serv Res.

[71] Bates DW, Singh H (2018). Two decades since to err is human: An assessment of progress and emerging priorities in patient safety. Health Aff.

[72] Luca CE, Sartorio A, Bonetti L (2024). Interventions for preventing and resolving bullying in nursing: A scoping review. Healthcare.

